# Awareness, susceptibility, and use of oral nicotine pouches and comparative risk perceptions with smokeless tobacco among young adults in the United States

**DOI:** 10.1371/journal.pone.0281235

**Published:** 2023-01-30

**Authors:** Meghan E. Morean, Krysten W. Bold, Danielle R. Davis, Grace Kong, Suchitra Krishnan-Sarin, Deepa R. Camenga

**Affiliations:** 1 Department of Psychiatry, Yale School of Medicine, New Haven, CT, United States of America; 2 Department of Emergency Medicine, Yale School of Medicine, New Haven, CT, United States of America; University of Technology Sydney, AUSTRALIA

## Abstract

**Background:**

Oral nicotine pouches (NPs) that contain nicotine but no tobacco leaves are rapidly gaining popularity. However, there is limited research on NPs, including within priority populations. In the current study, we examined awareness of, susceptibility to, and use of NPs in young adults as well as comparative risk perceptions with smokeless tobacco.

**Methods:**

In 2021, 609 young adults (18–25 years) completed an online survey. Participants reported on NP awareness, susceptibility, and use as well as on comparative product perceptions for NPs versus smokeless tobacco. We ran unadjusted between-groups comparisons and an adjusted multinomial logistic regression to identify relationships between product perceptions and NP susceptibility and use.

**Results:**

41.5% of participants had heard of NPs before. Participants were non-susceptible (66.2%), susceptible (23.5%), or had used NPs (10.3%). Comparative product perceptions between NPs and smokeless tobacco suggested that young adults, as a whole, expressed uncertainty about the relative risk/benefit of using NPs versus smokeless tobacco. However, as expected, unadjusted and adjusted findings indicated that favorable perceptions of NPs versus smokeless tobacco were disproportionately observed among susceptible participants and NP users compared to non-susceptible individuals. Demographic differences were also observed (e.g., NP users were more likely than non-susceptible and susceptible individuals to have used smokeless tobacco).

**Conclusions:**

Young adults reported awareness of, susceptibility to, and use of NPs, with findings indicating that favorable perceptions of NPs versus smokeless tobacco may contribute to NP susceptibility and use beyond known correlates like smokeless tobacco use. However, further research is needed to understand the full range of factors that are associated with NP susceptibility and use. It will be important to disentangle factors that are associated with potential positive public health impacts (e.g., switching from smokeless tobacco to exclusive NP use) from those associated with negative public health impacts (e.g., initiation among nicotine naïve individuals).

## Introduction

In 2016, oral nicotine pouches (NPs)—small, fiber pouches that contain nicotine but no tobacco leaf [1]—entered the commercial market of the United States (U.S.). NPs are placed between the gum and the lip where nicotine is absorbed orally, similar to traditional smokeless tobacco products. Research indicates that NPs contain a similar amount of nicotine as do traditional smokeless tobacco products [2] but with dramatically fewer harmful chemicals [3]; although independent research is required, an industry-sponsored study reported that NPs have a toxicant profile that is similar to nicotine replacement therapies that are approved for tobacco cessation [3]. The ability of NPs to provide sufficient nicotine to prevent withdrawal [2] combined with a potentially favorable toxicant profile [3] may benefit public health if people switch from traditional smokeless tobacco and/or combustible tobacco product use [4]. Use of NPs may be especially attractive for people who use smokeless tobacco—the majority of current NP users [5]. However, NPs also could contribute to public health harm if they become popular among tobacco naïve individuals or vulnerable populations, including adolescents and young adults (AYAs). Prevalence rates for NP use among AYAs have varied across studies. In 2021, estimates of having ever used NPs were 3.0% among high school students participating in the 2021 National Youth Tobacco Survey [6] and 5.2% among young adults (18–20 years) participating in the industry-sponsored 2021 Altria Client Services Underage Tobacco Use Survey [7]. However, estimates of past-month NP use among AYAs (15–24 years) have been as high as 13%, as reported by the Truth Initiative in 2021 [8].

Given that NPs are regulated by the United States Food and Drug Administration, which is charged with regulating tobacco products to protect public health, it is important not only to understand rates of NP use among various populations of interest (e.g., people who currently use smokeless tobacco, vulnerable populations like AYAs) but also precursors to use including product awareness and susceptibility (i.e., the lack of a clear resolution not to use a product; [9–12]). Assessing public perceptions about NPs, including comparative risk perceptions with similar tobacco products like smokeless tobacco, also is important as favorable perceptions may relate to both NP susceptibility and use. Obtaining initial evidence about who is using NPs, who may be attracted to future use, and how risk perceptions may play a role in susceptibility and use is critical for building our foundational understanding of NPs as an emerging nicotine product. Study findings can serve as an impetus for future research, including assessing motives for using NPs (e.g., switching from more dangerous products like cigarettes or smokeless tobacco; hiding nicotine product use due to the concealability of NPs) and identifying factors that impact susceptibility (e.g., appealing marketing tactics). This more nuanced future research can establish the relative negative versus positive impacts of NPs on public health, and, if necessary, guide the development of future prevention efforts and regulatory actions to protect vulnerable populations.

For the current study, we surveyed U.S. young adults (ages 18–25 years) about their awareness of, susceptibility to, and use of NPs. We also assessed 13 comparative product perceptions for NPs versus smokeless tobacco. We used smokeless tobacco as the comparator given that NPs and smokeless tobacco both deliver nicotine orally and published research exists on the relative amounts of nicotine and other harmful chemicals present in these two product types (e.g., there are more harmful chemicals in smokeless tobacco) [2, 3].

We expected to observe significant, unadjusted between-groups differences for each of the 13 comparative perception items, such that young adults who were susceptible to using NPs or who had used NPs, compared to non-susceptible individuals, would perceive NPs as disproportionately safer/better than smokeless tobacco. Further, we expected NP susceptibility and use would continue to be associated with holding more favorable views of NPs relative to smokeless tobacco even after accounting for demographic covariates known to relate to tobacco product use as well as actual use of tobacco products including e-cigarettes, cigarettes, hookah, cigars/cigarillos, and smokeless tobacco. Based on prior work showing that most adults who use NPs currently use or previously used smokeless tobacco [5], we hypothesized that smokeless tobacco use would be associated with susceptibility to and use of NPs. Finally, we expected males to be more likely than females to be susceptible to NPs or to have used NPs based on research indicating that males are more likely than females to use smokeless tobacco [13, 14] and nicotine pouches [5, 6, 15]. While research explaining sex differences in nicotine pouch use is lacking, cultural factors (e.g., masculinity, rurality, rebelliousness, participation in athletics) largely are thought to account for the observed sex differences in using smokeless tobacco [14].

## Materials and methods

### Participants and procedure

All study procedures were approved by the Yale School of Medicine Institutional Review Board. From September through October 2021, 1,239 U.S. young adults (ages 18–25 years) participated in a 20-minute, anonymous online survey via Qualtrics^TM^ panels. Qualtrics Online Sample, a secure market research service operated by *Qualtrics*, *Inc*, recruited and compensated all participants. All eligible individuals were directed to an online consent form, and consent was required prior to completing the remainder of the survey. To maintain anonymity, all participants provided consent by checking a box that indicated their desire to participate.

The primary aim of the parent study was to examine risk perceptions about tobacco-free e-cigarettes, so we used quota sampling procedures to purposefully oversample young adults who use e-cigarettes: exclusive e-cigarette use (n = 334), use of both e-cigarettes and other tobacco product(s) (n = 334), exclusive use of tobacco product(s) other than e-cigarettes (n = 206), and no use of tobacco products (n = 365).

To reduce participant burden, we randomized approximately 50% of the total sample (n = 609) to a condition that required them to report on comparative perceptions between NPs and smokeless tobacco. These individuals comprise the analytic sample for the current study. Participants were provided with the following description of NPs: “Nicotine pouches are small, white, pre-portioned pouches that you put in your mouth and tuck between your cheek and your gums. Unlike smokeless tobacco products (e.g., chew, dip, snus, and snuff) that contain tobacco leaves, nicotine pouches do not contain any actual tobacco leaves. Therefore, nicotine pouches often are marketed as tobacco-free by the companies that make them. The nicotine in these pouches either comes from purifying the nicotine from tobacco plants (i.e., is tobacco-derived) or is created synthetically (artificially) in a lab using chemicals that do not come from tobacco plants.” Participants were provided with an image that provided brand examples of NPs, some of which contain tobacco-derived nicotine (e.g., Zyn, On!) and some which contain synthetic nicotine (e.g., Fre, Niin).

After reading the definition, participants reported on comparative product perceptions associated with using NPs versus smokeless tobacco. We developed our product perception items based on the Smokeless Tobacco Expectancies Scale [16] (e.g., addiction, gum disease, teeth staining) and manufacturer claims about nicotine pouches (e.g., good flavors, smooth taste; all items and the response scale are presented in Table 2). After reporting on comparative product perceptions, all participants reported on NP awareness: “Before today, had you ever heard of tobacco-free nicotine pouches?” (no/yes). Participants who were aware of NPs were asked if they had “ever used a tobacco-free nicotine pouch” (no/yes). Participants who had never heard of or had never used NPs were queried about susceptibility: “At any time in the next year, do you think you will use a tobacco-free nicotine pouch?” (Definitely not, probably not, probably yes, definitely yes). We coded young adults as susceptible if they answered anything other than “definitely not” [12].

### Additional measures

#### Screening measures

Participants were asked if they had ever used each of the following products (no/yes): disposable pod vape, e-hookah, cig-a-like, vape pen, JUUL, a rechargeable pod device other than JUUL, Mod/APV, hookah, cigar/cigarillo, smokeless tobacco, and nicotine pouch. We included a brief description and an image for each product. Participants who endorsed use of any of the e-cigarette devices were coded as having ever used e-cigarettes (i.e. “Ever Use”).

#### Demographics

We assessed participant age (# years), biological sex (male/female), Hispanic ethnicity (no/yes), race (White, Black, Asian, American Indian or Alaska Native, Pacific Islander or Native Hawaiian, Other [write-in]), and subjective financial situation [17] as a proxy for socioeconomic status. Based on the limited cell sizes for some racial minority groups, we created a variable reflecting White, Black, and Other Race for inclusion in the analyses.

### Analytic plan

#### Product perceptions

Participants who had never used NPs reported on susceptibility irrespective of whether they had heard of NPs prior to the study or whether they heard of NPs for the first time after reading the description provided to them in the study. To determine whether non-susceptible and susceptible individuals, respectively, should be separated into unique categories for statistical analyses based on prior awareness of NPs (no/yes), we conducted two sets of independent samples t-tests (after first checking the data for normality). In the first set of tests, we compared the product perception item means of non-susceptible individuals who had prior awareness of NPs versus those who did not. A second set of tests was conducted to compare item means of susceptible individuals who had prior awareness of NPs versus those who did not. If no differences were observed, those with and without prior awareness of NPs would be combined into two categories: not susceptible to NPs and susceptible to NPs. However, if significant differences were observed, we would create up to five mutually exclusive groups for analyses (i.e., never heard of NPs and not susceptible, heard of NPs and not susceptible, never heard of NPs and susceptible, heard of NPs and susceptible, and ever used NPs).

After determining the appropriate number of participant groups, a one-way ANOVA was run to compare unadjusted product perceptions for NPs versus smokeless tobacco based on susceptibility and NP use. A correction for multiple comparisons (i.e., Tukey) was applied. All analyses described in this section were conducted using SPSS version 28.

#### Evaluating the latent structure of product perceptions

There was overlap in the content of the product perception items (e.g., NPs are less harmful to a person’s health; NPs are less harmful to a person’s heart). To avoid issues with multicollinearity in the regression-based modeling described below, we ran a factor analysis within Mplus 8.1 to establish whether we could score the product perception items unidimensionally. If so, we planned to include a single score for comparative product perceptions in the multinomial logistic regression model described below.

#### Examining correlates of NP susceptibility and use

Using SPSS version 28, we conducted a multinomial logistic regression model in which participant demographics (i.e., age, sex, Hispanic ethnicity, race, subjective financial status); ever use of e-cigarettes, cigarettes, hookah, cigars/cigarillos, and smokeless tobacco; and product perceptions of NPs versus smokeless tobacco were included as predictors of NP susceptibility and NP ever use. To examine comparisons between all three NP use groups (i.e., not susceptible, susceptible, ever use), model results are presented when those were not susceptible to NP use served as the reference group (i.e., versus those who were susceptible and those who had used NPs) and when those who were susceptible to NP use served as the reference group (i.e., versus those who had used NPs).

## Results

### Descriptive statistics

Descriptive statistics for all study variables are included in Table 1. Note that there were no missing data. In brief, participants were 55.8% female, 36.0% Hispanic, 53.5% white, and had an average age of 21.24 (2.30) years. While the rate of ever NP use (10.3%) was lower than the rates of ever use for all other tobacco products (range: smokeless tobacco [13.8%] to e-cigarettes [75.2%]), 41.5% of participants had heard of NPs and 23.5% were susceptible to future use.

**Table 1:** Descriptive statistics.

Age	21.24 (2.30)
Female Sex	55.8
Hispanic	36.0
Race	
White	53.5
Black	22.5
Asian	6.9
Other	17.1
Subjective Financial Status	2.54 (0.98)
Tobacco Product Use (Ever)	
E-cigarettes	75.2
Cigarette	44.0
Hookah	33.0
Cigar/Cigarillo	36.1
Smokeless Tobacco	13.8
Nicotine Pouches	
Awareness	41.5
Susceptibility	23.5
Ever Use	10.3
Average Product Perception Score (Nicotine Pouch vs Smokeless)	2.73 (0.74)

N = 609; Mean (standard deviation) is presented for continuous variables and percent is presented for categorical variables. Scoring for Subjective Financial Status (1 = I don’t meet basic expenses, 2 = I just meet basic expenses, 3 = I meet needs with a little left over, and 4 = I live comfortably; range 1–4); Scoring for Average Risk Scores (range 1 [strongly disagree] - 5 [strongly agree]).

### Product perceptions

Data were deemed to sufficiently approximate normality based on skewness and kurtosis values across all product perception items (maximum value for skewness = -0.41; maximum value for kurtosis = -0.82), thus supporting the planned analyses. For individuals reporting susceptibility to NPs or no susceptibility to NPs, respectively, the independent samples t-tests revealed no significant differences in product perception item means based on having prior awareness of NPs (S1 Table). Thus, we created a three-level variable with mutually exclusive categories reflecting individuals who were not susceptible to NPs (66.2%, n = 403), were susceptible to NPs (23.5%, n = 143), or had used NPs (10.3%, n = 63).

Mean scores for the each of the individual product perception items comparing NPs to smokeless tobacco fell within the mid-range of the scale, and the median and mode for each item and for full scale score were 3 (i.e., “neither disagree nor agree”; Table 2). When comparing perceptions across NP status (i.e., not susceptible, susceptible, use), with the exception of the perception that NPs have more of a chemical taste than does smokeless tobacco, young adults who were susceptible to NPs universally reported disproportionately stronger favorable perceptions for NPs relative to smokeless tobacco for all items compared to those who were not susceptible to NP use (Table 2). Compared to young adults who were not susceptible to NP use, young adults who had used NPs reported stronger favorable perceptions for NPs relative to smokeless tobacco for all items. The perceptions held by individuals who had used NPs did not differ from those who were susceptible to using NPs.

**Table 2:** Comparative product perceptions for nicotine pouch versus smokeless tobacco use.

	Nicotine Pouch Status		
Compared to smokeless tobacco, nicotine pouches…	Not Susceptible	Susceptible	Ever Use
Are less harmful to a person’s health***	2.32 (1.13)^A^	2.92 (1.08)^B^	2.78 (1.31)^B^
Are less harmful to a person’s heart***	2.34 (1.07)^A^	2.87 (1.07)^B^	2.67 (1.14)^B^
Are less harmful to a person’s mouth or gums***	2.28 (1.12)^A^	2.77 (1.19)^B^	2.71 (1.31)^B^
Are less likely to stain your teeth***	2.54 (1.16)^A^	2.90 (1.14)^B^	3.21 (1.32)^B^
Are less addictive***	2.29 (1.11)^A^	2.81 (1.06)^B^	2.63 (1.13)^B^
Are less expensive***	2.63 (0.91)^A^	2.99 (1.04)^B^	3.06 (1.09)^B^
Are easier for a person my age to purchase**	2.76 (1.03)^A^	3.08 (1.06)^B^	3.14 (1.13)^B^
Taste less like tobacco***	2.78 (1.01)^A^	3.13 (1.03)^B^	3.37 (1.29)^B^
Taste smoother***	2.66 (0.95)^A^	3.10 (1.09)^B^	3.02 (1.24)^B^
Have flavors that taste better***	2.69 (0.98)^A^	3.13 (1.08)^B^	3.30 (1.21)^B^
Taste cleaner***	2.68 (0.97)^A^	3.13 (1.05)^B^	3.37 (1.31)^B^
Have more of a chemical taste**	2.91 (0.95)^A^	3.04 (0.93)^A,B^	3.30 (1.12)^B^
Taste better overall***	2.65 (0.99)^A^	3.09 (1.07)^B^	3.24 (1.20)^B^
Perceptions Total Score***	2.58 (0.73)^A^	3.00 (0.62)^B^	3.06 (0.75)^B^

N = 609; Not Susceptible (n = 403); Susceptible (n = 143); Ever Use (n = 63)

** *p* < .01

*** *p* < .001

*p*-values reflect the overall significance of each ANOVA model. Within a row, superscript letters that differ from one another indicate a significant between groups difference at *p* < .05 with a Tukey correction for multiple comparisons applied.

### Evaluating the latent structure of product perceptions

Results of the factor analysis supported scoring the 13 product perceptions as a unidimensional scale (S2 Table). As such, a mean scale score for product perceptions was included in the multinomial regression analysis examining adjusted correlates of NP susceptibility and use.

### Correlates of NP susceptibility and use

Compared to young adults who were not susceptible to NPs: 1) susceptible young adults were significantly more likely to be male (OR_adj_ = 1.52), to have used e-cigarettes (OR_adj_ = 2.95), and to have more favorable perceptions of NPs relative to smokeless tobacco (OR_adj_ = 2.54), and 2) young adults who had used NPs were significantly more likely to be younger (OR_adj_ = 0.84), to have used e-cigarettes (OR_adj_ = 6.90), cigars/cigarillos (OR_adj_ = 2.88), and smokeless tobacco (OR_adj_ = 16.45), and to report more favorable perceptions of NPs relative to smokeless tobacco (OR_adj_ = 2.18; Table 3). Finally, compared to young adults who were susceptible to NPs, those who had used NPs were significantly younger (OR_adj_ = 0.78) and more likely to have used cigars/cigarillos (OR_adj_ = 4.70) and/or smokeless tobacco (OR_adj_ = 20.10).

**Table 3:** Associations between demographics, tobacco use status, comparative risk perceptions and nicotine pouch status.

	Versus Not Susceptible to Nicotine Pouches												Versus Susceptible to Nicotine Pouches					
	Susceptible to Nicotine Pouches						Ever Use of Nicotine Pouches						Ever Use Nicotine Pouches					
	B	SE	Wald	ORadj	95% CI		B	SE	Wald	ORadj	95% CI		B	SE	Wald	ORadj	95% CI	
Age	0.08	0.05	2.65	1.08	0.98	1.19	-0.17	0.09	4.17	0.84*	0.71	0.99	-0.25	0.09	7.81	0.78**	0.65	0.93
Male	0.42	0.21	4.00	1.52*	1.01	2.29	0.14	0.37	0.15	1.15	0.56	2.36	-0.28	0.39	0.51	0.76	0.35	1.63
Hispanic	0.13	0.22	0.34	1.14	0.74	1.76	-0.34	0.39	0.78	0.71	0.33	1.52	-0.47	0.41	1.31	0.62	0.28	1.40
Race (Ref. White)																		
Black	0.26	0.26	1.03	1.30	0.79	2.14	-0.25	0.45	0.30	0.78	0.33	1.88	-1.01	0.53	3.66	0.36	0.13	1.02
Other Race	0.30	0.27	1.27	1.35	0.80	2.27	-0.71	0.50	1.99	0.49	0.18	1.32	-0.50	0.47	1.13	0.60	0.24	1.53
Financial Status	-0.14	0.11	1.56	0.87	0.71	1.08	0.25	0.18	1.79	1.28	0.89	1.83	0.38	0.20	3.77	1.46	1.00	2.15
Ever Use																		
E-cigarette	1.08	0.28	14.57	2.95***	1.69	5.14	1.93	0.85	5.12	6.90*	1.30	36.73	0.85	0.88	0.93	2.34	0.42	13.17
Cigarette	0.28	0.23	1.47	1.32	0.84	2.06	0.52	0.42	1.53	1.68	0.74	3.83	0.24	0.45	0.30	1.28	0.53	3.06
Hookah	-0.12	0.25	0.23	0.89	0.54	1.45	0.39	0.40	0.95	1.48	0.67	3.23	0.51	0.43	1.39	1.66	0.72	3.87
Cigar/cigarillo	-0.49	0.26	3.53	0.61	0.37	1.02	1.06	0.42	6.38	2.88*	1.27	6.55	1.55	0.45	11.63	4.70***	1.93	11.42
Smokeless	-0.20	0.41	0.24	0.82	0.37	1.82	2.80	0.39	51.91	16.45***	7.68	25.24	3.00	0.47	40.40	20.10***	7.97	50.69
Product Perceptions	0.93	0.17	29.91	2.54***	1.82	3.56	0.78	0.27	8.58	2.18**	1.29	3.66	-0.16	0.29	0.30	0.86	0.49	1.49

Note.

* *p* < .05

** *p* < .01

*** *p* < .001.

Variables included in the model were: age, sex, Hispanic ethnicity, race, subjective financial status; ever use of e-cigarettes, cigarettes, hookah, cigars/cigarillos, and/or smokeless tobacco; and product perceptions of nicotine pouches versus smokeless tobacco. Age, financial status, and product perceptions were scored such that observed positive relationships with nicotine pouch status (i.e., an adjusted odds ratio < 1) reflect older age, higher financial status, and more positive product perceptions. Conversely, negative relationships with nicotine pouch status (i.e., an adjusted odds ratio < 1) reflect younger age, lower financial status, and less favorable product perceptions. For the columns titled “Versus Not Susceptible to Nicotine Pouches,” those who were not susceptible to nicotine served as the reference group. For the column titled “Versus Susceptible to Nicotine Pouches,” those who were susceptible to nicotine pouches served as the reference group. B (regression coefficient), SE (standard error of the regression coefficient), Wald (Wald chi-square value), ORadj (adjusted odds ratio), CI (Confidence Interval).

## Discussion

The current study uniquely examined young adults’ comparative product perceptions for nicotine pouches versus smokeless tobacco. The rate of awareness of NPs observed in the current study (41.5%) was in line with estimates observed in a recent study of youth and young adults (ages 13–20 years; range for rates of awareness: 40–50%) [7]. The rate of susceptibility (23.5%) observed in our sample was considerable and was higher than what was observed recently among adults who smoke cigarettes (susceptibility = 16.8%) [18]. It merits noting, however, that the time frames used to assess susceptibility differed across these studies; the current study assessed expectations for trying NPs in the next year while the study of adults who smoke [18] assessed interest in trying NPs within the next 6 months. Finally, the rate of NP use in the current sample (10.3%) was higher than what has been observed recently for adults who smoke cigarettes (5.6%) [18], high school students (3.0%) [6], and young adults ages 18–20 years (5.2%) [7]. These findings may be driven by a range of factors that were not assessed in the current study including a continued increase in the popularity of NPs overall and/or more frequent or more targeted marketing to young adults. Thus, more research in this area is needed. However, it also is possible that the elevated rates of susceptibility and use observed in our sample relative to the AYA samples described above may be linked to the fact that we oversampled young adults who currently used tobacco products for this study; in prior research with young adults, increased willingness to try NPs was observed among those who currently used combustible and non-combustible tobacco products relative to those who did not use any tobacco products [19].

With regard to comparative product perceptions for NPs versus smokeless tobacco, mean scores for the items and the unidimensionally scored scale were in the mid-range of the scale, and both median and mode responses were “neither disagree nor agree.” These findings appear to reflect uncertainty about the relative risk/appeal of NPs versus smokeless tobacco in the sample as a whole. This finding aligns with prior research suggesting that young adults are uncertain about the risks of using NPs relative to cigarettes and e-cigarettes [19]. However, when unadjusted differences were examined by NP status (i.e., not susceptible, susceptible, use), where significant differences emerged, stronger beliefs that NPs are less harmful/better than smokeless tobacco were observed among young adults who were susceptible to NPs or who had used NPs relative to non-susceptible individuals. There were no differences in comparative product perceptions between young adults who were susceptible to NPs and those who had used NPs. Although no study of which we are aware has examined comparative product perceptions for NPs versus smokeless tobacco in young adults, the findings suggest that those who are drawn to or who have used NPs may be aware that nicotine pouches likely are less harmful than is smokeless tobacco [3], a tobacco product for which many significant risks (e.g., cancer, negative oral health outcomes [20]) are well-established.

When considering adjusted findings, compared to both non-susceptible and susceptible young adults, young adults who used NPs were more likely to be younger, to use cigars/cigarillos, and to use smokeless tobacco. Those who had used NPs also were more likely than non-susceptible individuals to use e-cigarettes and to perceive NPs as disproportionately safer/better than smokeless tobacco. Compared to non-susceptible young adults, susceptible young adults were more likely be male, to use e-cigarettes, and to perceive NPs as disproportionately safer/better than smokeless tobacco. Where significant findings emerged for sex, smokeless tobacco use, and comparative product perceptions, respectively, they were consistent with our hypotheses suggesting that males [5, 13–15], young adults who used smokeless tobacco [5], and those holding favorable views of NPs would be more likely to be susceptible to or to use NPs. However, in no case did group membership (e.g., being male, using smokeless tobacco products) significantly differentiate all groups (i.e., susceptible individuals from non-susceptible individuals, those who had used NPs from non-susceptible individuals, and those who had used NPs from susceptible individuals).

Several additional findings emerged for which we did not have a priori hypotheses. First, compared to non-susceptible individuals, susceptible young adults and those who had used NPs were more likely to use e-cigarettes. While additional research is needed to explain this finding, the observed relationships between e-cigarette use and both NP susceptibility and NP use may be linked to the oversampling of individuals who used e-cigarettes in our current sample or to the fact that both NPs and some brands of e-cigarettes are marketed as containing “tobacco-free” or synthetic nicotine. Second, young adults who had used NPs were more likely than non-susceptible and susceptible young adults to use cigars/cigarillos and to be younger. Both findings require additional study in larger, more representative samples, but the fact that young adults who had used NPs in our sample were the youngest of the three groups raises concerns about product accessibility, experimentation, and use among younger individuals.

Several study limitations must be considered when interpreting the findings. First, data were obtained from a convenience sample of young adult Qualtrics panelists in the United States, which may limit generalizability. Second, the study was cross-sectional, and future research is needed to examine causal relationships among the constructs assessed in the current study. Third, we assessed susceptibility using only one question that was focused on use in the upcoming year. However, prior work has suggested that including additional questions related to curiosity and the likelihood of use if a friend offered the product may more fully represent the construct of susceptibility [12, 21]. Fourth, young adults who currently used tobacco products were over-represented in our analytic sample based on the quota-based sampling we employed for the parent study. As such, rates of awareness, susceptibility, and use cannot be considered to represent the broader population and cannot be considered prevalence estimates. Because we did not set any quota based on NP use, the rates observed in the current study may be relatively representative of young adults who use tobacco products. However, additional research in nationally representative samples is needed to capture accurate prevalence of these constructs. Fifth, we focused on ever use of NPs and other tobacco products in the current study because focusing on past-month use would have reduced statistical power to detect effects and would have produced small, unstable cell sizes in our model. Future research examining relationships between current use of NPs and other tobacco products is needed. Sixth, in the description of NPs provided to participants we noted that NPs (as a product class) often are marketed as “tobacco-free” irrespective of the type of nicotine they contain (i.e., tobacco-derived versus synthetic) because NPs do not contain any tobacco leaves. Although several brands of NPs that contain synthetic nicotine have entered the market (e.g., Juice Heads, FRE, 2One), many pouch brands that contain tobacco-derived nicotine, including the most popular brand, Zyn [22], continue to be marketed as “tobacco-free.” As such, we chose to have our definition reflect current marketing practices for NPs. As a result, we were unable to differentiate between perceptions about, susceptibility to, and use of synthetic versus tobacco-derived nicotine pouches in the current study, although this has been examined elsewhere [23]. Seventh, we did not include a comparison category simply referred to as “nicotine pouches.” Based on e-cigarette research, including the term “tobacco free” in our definition may have decreased risk perceptions about NPs and/or increased use intentions in our young adult sample [24]. While we could not directly assess the impact of the term “tobacco-free” in the current study, future research is needed to establish the impact of this term on product perceptions, susceptibility, and use of NPs. Eighth, it is not clear the extent to which the correlates of NP susceptibility and use are unique to NPs or would generalize to smokeless tobacco, and future research is needed on this topic [21]. Finally, it is possible that NP susceptibility and use may be beneficial for young adults who are using higher-risk tobacco products (e.g., cigarettes, smokeless tobacco) and wish to switch to exclusive NP use as a means of harm reduction. However, we did not assess reasons for use or intentions to switch from more harmful products to NPs, so more nuanced research is needed to examine the extent to which susceptibility and use should be considered as increasing versus decreasing health risks in this population.

In sum, the present study contributes to the growing literature on NP awareness, susceptibility, and use among young adults. Ever use of NPs (10.3%) was lower than other tobacco products in the current sample, although the observed rate in our sample was higher than those observed in recent studies of youth [6], young adults [7], and adults who smoke cigarettes [18]. In addition, rates of NP awareness (41.5%) and susceptibility (23.5%) both were considerable. Comparative product perceptions between NPs and smokeless tobacco suggested that young adults, as a whole, expressed uncertainty about the relative risk/benefit of using NPs versus smokeless tobacco. However, there was variability in responses, with those who were susceptible to or who had used NPs expressing more favorable perceptions about NPs than non-susceptible individuals. Finally, where significant findings emerged, known risk factors for NP use (e.g., being male, using smokeless tobacco) in addition to holding positive perceptions of NPs relative to smokeless tobacco were disproportionately observed among young adults who were susceptible to or who had used NPs relative to non-susceptible individuals. In addition, several unique findings that require further assessment were observed (e.g., relationships between e-cigarette use, cigars, and NP susceptibility and use). When considered in concert, the findings suggest that young adults are aware of, are susceptible to, and are using NPs, and that comparative risk perceptions for NPs versus smokeless tobacco use may play an important role in susceptibility and use. Expanded monitoring is needed to fully understand factors that confer risk for NP use in young adults, and research should be expanded to assess NP-related constructs in other priority groups. Findings can be used to inform the FDA about the relative extent to which NPs are being used for harm reduction (e.g., to replace more harmful tobacco products like smokeless tobacco or cigarettes) and are leading to initiation of new use among vulnerable populations like youth and young adults.

## Supporting information

S1 Table Examining potential between-groups differences in product perceptions based on susceptibility and prior awareness of nicotine pouches. (DOCX)

S2 Table Factor analysis supporting scoring comparative product perceptions for nicotine pouches versus smokeless tobacco as a unidimensional scale. (DOCX)

S1 Data (XLSX)
