# Supplementary material for: Awareness, susceptibility, and use of oral nicotine pouches and comparative risk perceptions with smokeless tobacco among young adults in the United States
Source: PLoS One. 2023 Jan 30;18(1):e0281235. doi: 10.1371/journal.pone.0281235 (PMC9886243; doi:10.1371/journal.pone.0281235)
Supplement: S1 Table — (DOCX) [file pone.0281235.s001.docx]

S1 Table. Examining potential between-groups differences in product perceptions based on susceptibility and prior awareness of nicotine pouches

|  |  |  |  |  |  |  |  |
| --- | --- | --- | --- | --- | --- | --- | --- |
|  |  | Nicotine Pouch (NP) Status | | | | | |
|  |  | Not Susceptible | | | Susceptible | | |
| Compared to smokeless tobacco, nicotine pouches |  | Never Heard of NPs |  | Heard of NPs | Never Heard of NPs |  | Heard of NPs |
| Are less harmful to a person's health |  | 2.28 (1.12) |  | 2.39 (1.14) | 2.87 (1.00) |  | 2.98 (1.19) |
| Are less harmful to a person’s heart |  | 2.29 (1.07) |  | 2.45 (1.08) | 2.72 (0.98) |  | 3.10 (1.17) |
| Are less harmful to a person’s mouth or gums |  | 2.28 (1.09) |  | 2.27 (1.19) | 2.58 (1.12) |  | 3.05 (1.23) |
| Are less likely to stain your teeth |  | 2.54 (1.16) |  | 2.54 (1.16) | 2.84 (1.03) |  | 3.00 (1.28) |
| Are less addictive |  | 2.29 (1.21) |  | 2.28 (1.09) | 2.79 (0.98) |  | 2.84 (1.18) |
| Are less expensive |  | 2.58 (0.91) |  | 2.71 (0.92) | 2.91 (0.96) |  | 3.01 (1.14) |
| Are easier for a person my age to purchase |  | 2.70 (1.02) |  | 2.89 (1.06) | 3.06 (1.04) |  | 3.10 (1.10) |
| Taste less like tobacco |  | 2.74 (1.00) |  | 2.85 (1.03) | 2.95 (1.07) |  | 3.38 (0.94) |
| Taste smoother |  | 2.62 (0.95) |  | 2.76 (0.94) | 3.13 (1.00) |  | 3.07 (1.23) |
| Have flavors that taste better |  | 2.65 (0.95) |  | 2.77 (1.03) | 3.12 (0.99) |  | 3.16 (1.21) |
| Taste cleaner |  | 2.66 (0.97) |  | 2.73 (0.97) | 3.09 (1.02) |  | 3.17 (1.11) |
| Have more of a chemical taste |  | 2.89 (0.95) |  | 2.95 (0.96) | 3.05 (0.89) |  | 3.03 (0.99) |
| Taste better overall |  | 2.61 (0.97) |  | 2.73 (1.01) | 3.04 (1.09) |  | 3.17 (1.06) |
| Perceptions Total Score |  | 2.55 (0.73) |  | 2.64 (0.74) | 2.93 (0.61) |  | 3.09 (0.62) |
| N = 609; Not Susceptible, Never Heard of NPs (n = 271); Not Susceptible, Heard of NPs (n = 132); Susceptible, Never Heard of NPs (n = 85); Susceptible, Heard of NPs (n = 58); An adjusted alpha value of 0.004 was used as the threshold for statistical significance (i.e., 0.05/14). Independent samples t-tests were used to compare the item means and the mean subscale scores for all perception items of non-susceptible individuals who had prior awareness of NPs versus those who did not. A second set of t-tests was used to compare item means and the subscale scores of susceptible individuals who had prior awareness of NPs versus those who did not. No significant differences were observed. | | | | | | | |
|  |  |  |  |  |  |  |  |
