# Supplementary material for: Awareness, susceptibility, and use of oral nicotine pouches and comparative risk perceptions with smokeless tobacco among young adults in the United States
Source: PLoS One. 2023 Jan 30;18(1):e0281235. doi: 10.1371/journal.pone.0281235 (PMC9886243; doi:10.1371/journal.pone.0281235)
Supplement: S2 Table — (DOCX) [file pone.0281235.s002.docx]

S Table 2. Factor analysis supporting scoring comparative product perceptions for

nicotine pouches versus smokeless tobacco as a unidimensional scale.

|  | Compared to smokeless tobacco, tobacco-free nicotine pouches... |
| --- | --- |
| **Model Fit Indices** |  |
| Root Mean Square Error of Approximation | 0.051 |
| Bentler's Comparative Fit Index | 0.956 |
| Standardized Root Mean Square Residual | 0.049 |
| **Factor Loadings** |  |
| Are less harmful to a person's health | 0.67 |
| Are less harmful to a person’s heart | 0.64 |
| Are less addictive | 0.57 |
| Are less expensive | 0.58 |
| Are easier for a person my age to purchase | 0.55 |
| Taste less like tobacco | 0.66 |
| Taste smoother | 0.75 |
| Have flavors that taste better | 0.70 |
| Taste cleaner | 0.73 |
| Have more of a chemical taste | 0.55 |
| Taste better | 0.75 |
| Are less likely to stain your teeth | 0.68 |
| Are less harmful to a person’s mouth or gums | 0.67 |
